# Intrathecal Baclofen in Children with Cerebral Palsy: A Critical Review of Selection Criteria, Rehabilitation Goals, Outcomes, and Complications

**DOI:** 10.3390/jcm15114091

**Published:** 2026-05-25

**Authors:** Natalia Retkowska-Tomaszewska, Piotr Defort, Anna-Maria Barciszewska, Dariusz Patkowski

**Affiliations:** 1 Division of Neurosurgery, Department of Pediatric Surgery, Traumatology and Urology, Karol Jonscher Clinical Hospital, 60-572 Poznan, Poland; 2 Department of Pediatric Surgery, Traumatology and Urology, Institute of Pediatrics, Poznan University of Medical Sciences, 60-572 Poznan, Poland; 3 Department of Nervous System Diseases, Collegium Medicum, University of Zielona Góra, 65-046 Zielona Góra, Poland; p.defort@cm.uz.zgora.pl; 4 Neurosurgery Center, Karol Marcinkowski University Hospital, 65-046 Zielona Góra, Poland; 5 Intraoperative Imaging Unit, Department of Neurosurgery, Institute of Nervous System Diseases, Poznan University of Medical Sciences, 60-355 Poznan, Poland; ambarciszewska@ump.edu.pl; 6 Department of Neurosurgery, University Clinical Hospital, 60-355 Poznan, Poland; 7 Department of Pediatric Surgery and Urology, Wroclaw Medical University, 50-556 Wroclaw, Poland; dariusz.patkowski@umw.edu.pl

**Keywords:** cerebral palsy, intrathecal baclofen, spasticity, neurorehabilitation, functional outcomes

## Abstract

**Background**: Spasticity is a major contributor to pain, impaired mobility, contractures, and caregiver burden in children with cerebral palsy. Intrathecal baclofen (ITB) is an established treatment for severe generalized spasticity when rehabilitation, oral medications, and focal interventions are insufficient or poorly tolerated. **Methods**: This critical review synthesizes current evidence on ITB in children with cerebral palsy, focusing on patient selection, screening, rehabilitation goals, functional outcomes, complications, and long-term management. **Results**: Available evidence consistently demonstrates substantial and sustained tone reduction with ITB, with associated improvements in comfort, positioning, ease of care, pain, and selected quality-of-life domains. However, gains in gross motor function are variable and depend on baseline motor phenotype, individualized treatment goals, and careful dose titration. Device-related complications, infections, catheter dysfunction, overdose, and withdrawal remain clinically significant risks requiring specialized multidisciplinary follow-up. Compared with selective dorsal rhizotomy and botulinum toxin injections, ITB provides a reversible and programmable option particularly suited to children with severe, generalized spasticity and high caregiving needs. **Conclusions**: ITB represents an important component of comprehensive, goal-directed spasticity management in appropriately selected children. Further high-quality longitudinal and comparative studies are needed to define long-term functional and cost-effectiveness outcomes better.

## 1. Introduction

Cerebral palsy (CP) is the leading cause of persistent physical disability in childhood and is commonly associated with spasticity, pain, musculoskeletal deformity, and reduced participation. In high-income countries, its prevalence has decreased by up to 40%, from 2.1 to 1.6 per 1000 live births, although rates remain higher in low- and middle-income countries [1,2]. Pediatric cerebral palsy results from abnormal development of, or injury to, the parts of the brain that control movement, balance, and posture. The disorder most often arises during pregnancy, but it may also occur during childbirth or shortly thereafter. The cause is frequently unknown. Risk factors include preterm birth, multiple gestation, certain maternal infections or toxic exposures during pregnancy, difficult delivery, and head trauma in early childhood [2,3]. In addition, inherited genetic factors may account for a substantial proportion of cases [4].

Spasticity is the most common motor manifestation, affecting approximately 70–80% of children with CP [1]. When severe and generalized, it interferes with positioning, hygiene, sleep, comfort, mobility, and caregiver burden. Although physiotherapy, oral antispastic agents, and focal treatments such as botulinum toxin remain first-line approaches, these measures are often insufficient in children with complex, multi-segmental spasticity [1,5,6]. Intrathecal baclofen (ITB) therapy was developed to deliver baclofen directly into the cerebrospinal fluid, achieving effective spinal cord concentrations with substantially lower systemic exposure than oral treatment [7,8,9]. In contemporary practice, the value of ITB lies not only in tone reduction but also in its potential to improve comfort, facilitate care, support rehabilitation, and help achieve individualized rehabilitation goals [10,11].

Although spasticity is common in CP, the clinically relevant target population for ITB is narrower. It consists mainly of children with severe generalized or multi-segmental hypertonia, often corresponding to Gross Motor Function Classification System (GMFCS) levels IV–V, although selected children at GMFCS level III may also be considered [1,11,12]. Population-based studies indicate that approximately one-quarter to one-third of children with CP may be classified at GMFCS levels IV–V, but only a subset develop refractory generalized spasticity severe enough to justify pump implantation [12,13,14]. Therefore, the indication for ITB should not be based on epidemiological severity or tone scores alone, but on the interaction between tone distribution, pain, positioning, hygiene, sleep, orthopedic burden, mobility, caregiver burden, and response to previous conservative and focal treatments [7,10,11].

For clinical decision-making, refractory spasticity may be defined as persistent, clinically meaningful hypertonia that continues to interfere with comfort, care, hygiene, sleep, positioning, transfers, mobility, rehabilitation participation, or pain control despite optimized conservative management. This includes adequate physiotherapy and postural management, treatment of aggravating factors such as pain, infection, constipation, hip pathology, or poor seating, oral antispastic medication when tolerated, and focal treatment such as botulinum toxin when the distribution of spasticity is appropriate [1,5,6]. Refractoriness should not be defined by a single Ashworth Scale (AS) or Modified Ashworth Scale (MAS) threshold alone, because tone scores do not reliably capture functional burden, caregiver burden, pain, sleep disruption, or participation restriction [10,15,16].

The International Classification of Functioning, Disability and Health provides a useful conceptual framework for interpreting ITB outcomes. Reduction in tone represents an effect at the level of body functions and structures. In contrast, the clinically meaningful goals of ITB often lie at the level of activities and participation, including improved sitting tolerance, easier hygiene, reduced pain, better sleep, improved transfers, participation in rehabilitation, and reduced caregiver burden [1,17]. This distinction is important because a measurable reduction in spasticity does not necessarily translate into improved gross motor function or participation, particularly in children with severe motor impairment, fixed musculoskeletal deformity, or mixed spastic-dystonic tone patterns [7,10,11]. This review critically discusses the role of ITB in children with CP, focusing on patient selection, screening, expected outcomes, complications, and its place among other invasive spasticity treatments.

## 2. Methodology

The review was based on a structured narrative search of the literature. PubMed/MEDLINE and Scopus were searched for articles addressing intrathecal baclofen therapy in children with cerebral palsy, with particular emphasis on patient selection, screening procedures, pharmacological rationale, rehabilitation goals, functional outcomes, complications, long-term management, and comparison with alternative invasive spasticity treatments. The main search terms included “cerebral palsy”, “intrathecal baclofen”, “baclofen pump”, “pediatric spasticity”, “screening trial”, “test dose”, “Gross Motor Function Classification System”, “functional outcomes”, “selective dorsal rhizotomy”, “botulinum toxin”, “complications”, “caregiver burden”, and “quality of life”.

The search included peer-reviewed articles published in English, with priority given to systematic reviews, randomized and prospective studies where available, large retrospective cohorts, consensus statements, clinical guidelines, and recent clinically relevant reviews. Foundational historical studies were also included, as necessary, to describe the development of ITB therapy and its pharmacological rationale [7,9,10,18,19]. Articles focusing exclusively on adult spasticity, non-cerebral palsy etiologies without relevance to pediatric CP, technical case reports without broader clinical implications, and publications lacking sufficient clinical detail were excluded. Because this was a critical narrative review rather than a systematic review or meta-analysis, formal risk-of-bias scoring and quantitative data pooling were not performed. Instead, the evidence was synthesized thematically, with particular attention to areas of consistency, uncertainty, and practical relevance for clinical decision-making.

## 3. Pathophysiology of Spasticity in Cerebral Palsy

Spasticity is a major clinical problem in children with CP. It results from upper motor neuron injury and impaired balance between excitatory and inhibitory motor pathways, leading to disinhibition of spinal reflex circuits, exaggerated stretch reflexes, and velocity-dependent resistance to passive movement [20,21]. On neurological examination, it may present with increased tone, hyperactive tendon reflexes, clonus, and spasms. Spasticity should be distinguished from dystonia, which is characterized by sustained or intermittent involuntary muscle contractions and abnormal postures, and from rigidity, in which resistance to passive movement is not velocity-dependent [1,17].

A clinically important distinction should also be made between the dynamic neural component of spasticity and secondary biomechanical changes. Chronic hypertonia during growth may contribute to altered muscle growth, muscle shortening, reduced sarcomere length, increased connective tissue stiffness, fibrosis, tendon shortening, joint contractures, hip displacement, scoliosis, and pain [20,21,22]. These secondary changes may progressively limit the range of motion and function even if neural hyperexcitability is later reduced. ITB primarily modulates the neural component of hypertonia by enhancing inhibitory spinal signaling; it does not reverse established fixed contractures, bony deformity, hip dislocation, or structural scoliosis [7,19,23]. This distinction is central to patient selection and family counseling, because ITB may improve comfort, care, positioning, and dynamic tone, but orthopedic procedures may still be required when fixed deformity is the dominant clinical problem.

## 4. Spasticity Assessment Scales

The severity of spasticity is commonly assessed using the Ashworth Scale and the Modified Ashworth Scale (Table 1). These scales are simple, reproducible, and easy to administer. However, they primarily measure resistance to passive movement and do not reliably distinguish velocity-dependent spasticity from fixed contracture [15,24,25]. Their interpretation may also be affected by pain, anxiety, positioning, examiner technique, dystonia, and musculoskeletal stiffness. Therefore, AS and MAS scores should not be used as isolated determinants of ITB candidacy [10,16].

Although the AS and MAS remain useful for rapid clinical documentation, their limitations are particularly relevant in children with CP. Both scales assess resistance to passive movement and may overestimate spasticity when resistance is caused by contracture, dystonia, pain, or soft-tissue stiffness. Conversely, they may underestimate clinically important problems such as spasms, sleep disruption, hygiene difficulty, pain, positioning difficulties, or caregiver burden [15,16]. Therefore, a child should not be selected for ITB solely because of a high MAS score, nor excluded solely because tone scores are modest if generalized hypertonia causes substantial pain, care difficulties, or functional limitation [7,10,11].

For this reason, Ashworth-based scales should be combined with passive range-of-motion examination, orthopedic evaluation, goal-based functional measures, and assessment with the Tardieu or Modified Tardieu Scale (Table 2). The latter evaluates muscle response at different stretch velocities and helps distinguish dynamic spasticity from fixed shortening [16,26,27]. In particular, the relationship between the angle of catch during fast stretch and passive range during slow stretch provides clinically useful information about the dynamic versus fixed components of hypertonia. This distinction is important before ITB therapy, because dynamic spasticity may respond to baclofen, whereas fixed contracture may require orthopedic or rehabilitative management rather than escalation of antispastic treatment [1,7,19,21].

Assessment before ITB should therefore extend beyond tone scores and include validated measures of motor function, comfort, care, participation, pain, and caregiver burden. Depending on age, communication ability, and GMFCS level, useful tools may include the Gross Motor Function Classification System, Gross Motor Function Measure, Goal Attainment Scaling, Canadian Occupational Performance Measure, CPCHILD, CP QOL-Child, pain scales, sleep assessment, sitting and transfer evaluation, and caregiver-reported measures of ease of care [1,12,28,29,30,31]. Goal Attainment Scaling is particularly useful because it allows individualized goals to be defined before treatment and evaluated after screening or pump implantation [28,32].

## 5. Mechanism of Action and Rationale for Intrathecal Delivery

Baclofen is a gamma-aminobutyric acid type B (GABA-B) receptor agonist that reduces excitatory neurotransmitter release and enhances inhibitory modulation at the spinal level, thereby decreasing spasticity and pathologic reflex activity [33,34]. Its clinical utility in oral form is frequently limited by poor penetration across the blood–brain barrier and dose-dependent systemic adverse effects, including sedation, dizziness, hypotonia, and, in some patients, seizures or autonomic instability [5,34].

Intrathecal administration was developed to overcome these limitations by delivering baclofen directly into the cerebrospinal fluid, where effective spinal cord concentrations can be achieved at doses substantially lower than those required with oral treatment [7,8,9]. This allows more targeted control of severe spasticity while reducing systemic exposure and improving tolerability. In addition, programmable delivery permits dose adjustment according to individual clinical needs, including diurnal variation in tone, sleep-related discomfort, and rehabilitation demands [7,35].

The pharmacokinetics of ITB are clinically relevant. Baclofen distribution within the cerebrospinal fluid is not uniform. Its concentration is influenced by bolus dose, infusion rate, catheter-tip location, cerebrospinal fluid flow, patient size, posture, and time after administration [8,35,36]. A rostrocaudal concentration gradient may occur, meaning that catheter position can influence whether the clinical effect is more pronounced in the lower limbs, trunk, or more generalized patterns of hypertonia [19,36]. This helps explain why catheter-tip placement and individualized dose titration are important, particularly in children with mixed upper- and lower-limb involvement, scoliosis, previous spinal surgery, or altered cerebrospinal fluid dynamics, including ventriculoperitoneal shunt-related considerations [35,37,38].

These pharmacological properties make intrathecal baclofen particularly attractive in children with severe generalized spasticity who do not respond adequately to rehabilitation, oral medications, or focal interventions such as botulinum toxin injections [5,11].

## 6. Intrathecal Baclofen Therapy

Intrathecal baclofen therapy delivers baclofen directly into the cerebrospinal fluid through an implanted pump-catheter system, achieving therapeutic spinal concentrations at doses approximately 100-fold lower than those required with oral therapy [8,9]. The first successful use of ITB for severe spasticity was reported in 1985, and its application in children with cerebral palsy was described shortly thereafter [9,18]. Since then, ITB has become an established treatment for severe generalized spasticity in both pediatric and adult populations [7,39].

In contemporary pediatric practice, ITB should not be viewed solely as a method of reducing muscle tone. Rather, it is a goal-directed intervention intended to improve comfort, ease of care, positioning, sleep, pain, and, in selected cases, functional performance and participation [11,40]. Its reversibility and programmability distinguish it from other invasive spasticity treatments, particularly in children with evolving motor patterns, growth-related orthopedic complications, and changing rehabilitation needs [19,35].

### 6.1. Intrathecal Baclofen Test Dose Trial

A screening intrathecal test dose is generally recommended before permanent pump implantation to assess clinical responsiveness, identify adverse effects, and refine treatment goals [7,10,19]. However, the use and interpretation of screening trials vary across centers. Therefore, a positive or negative trial should not be interpreted mechanically. The most common approach is a single bolus injection by lumbar puncture, followed by serial assessment over several hours. Response is typically documented using tone measures such as the Ashworth Scale, Modified Ashworth Scale, or Tardieu/Modified Tardieu assessment. Still, the most clinically meaningful evaluation should also include comfort, pain, positioning, sleep tendency, transfers, sitting tolerance, hygiene, ease of care, and the child’s individualized goals [7,11,35].

The single-bolus test has practical advantages: it is relatively simple, short, and avoids temporary catheter placement. However, it may not fully reproduce the effects of chronic programmable infusion. A child may show limited response during a short observation window because of suboptimal dose, pain, anxiety, dystonia, fixed contracture, inadequate goal selection, or because the relevant outcome requires longer observation than a few hours [7,8,10]. Conversely, an apparently strong reduction in tone may be clinically undesirable if it compromises head control, sitting stability, standing, transfers, or gait, particularly in ambulatory or partially ambulatory children who use increased tone as a compensatory strategy [5,41]. For this reason, false-negative and false-positive interpretations are possible. A false-negative screening trial may occur when the test dose is too low, when assessment focuses only on limb tone rather than comfort or care, when fixed deformity masks reduction in dynamic tone, or when dystonia and fluctuating hypertonia complicate bedside evaluation [7,10,19]. In selected complex cases, prolonged observation or a continuous, temporary intrathecal infusion may be considered, as continuous trialing may more closely approximate the conditions of pump therapy. Nevertheless, such approaches are more invasive and require appropriate monitoring, experienced staff, and clear predefined outcome criteria [7,35]. The decision to proceed to implantation should therefore integrate trial response, treatment goals, risk profile, caregiver capacity, orthopedic status, and multidisciplinary consensus rather than relying on tone reduction alone.

### 6.2. Indications

ITB therapy is generally considered in children with cerebral palsy who have severe generalized or multi-segmental spasticity that interferes with comfort, hygiene, positioning, sleep, pain control, mobility, or caregiver burden, despite appropriate rehabilitation and medical management [10,11]. It may also be considered when oral antispastic medications are ineffective or poorly tolerated, or when focal treatments such as botulinum toxin injections are insufficient because the pattern of hypertonia is too diffuse [5,42].

In pediatric cerebral palsy, ITB is particularly relevant because uncontrolled spasticity during growth may contribute to progressive contractures, hip displacement, spinal deformity, impaired positioning, and reduced participation in rehabilitation and daily life [21,22]. Accordingly, the rationale for ITB extends beyond a simple reduction in tone and includes the prevention of secondary complications and the facilitation of long-term care.

### 6.3. Patient Selection

Appropriate patient selection is central to successful ITB therapy. The best candidates are not necessarily those with the highest tone scores, but those in whom tone reduction is most likely to translate into meaningful improvements in comfort, care, positioning, pain, sleep, rehabilitation participation, or selected functional goals [7,11]. This distinction is especially important in children with cerebral palsy, where therapeutic priorities may differ substantially according to motor phenotype, Gross Motor Function Classification System level, orthopedic status, and family expectations.

Pre-implantation evaluation should be multidisciplinary and should include input from rehabilitation medicine, neurology, neurosurgery, and, where relevant, orthopedics and allied health professionals [1,7]. Assessment should extend beyond spasticity scales to include pain, ease of care, sitting tolerance, transfers, sleep, communication, participation, and caregiver burden. Treatment goals must be realistic and explicitly discussed with families, particularly in ambulatory children, in whom excessive tone reduction may impair compensatory function rather than improve it [5,41].

Orthopedic and neurosurgical factors are also relevant. Scoliosis severity, hip status, previous spinal surgery, abdominal anatomy, and ventricular shunt status may all influence candidacy and operative planning [7,22,37]. Continuous ITB may be associated with progression of scoliosis in some patients, although causality remains difficult to establish, and early ITB has not been shown to prevent progression of hip displacement [22,23]. Epilepsy is not an absolute contraindication, but seizure history should be considered when balancing risks and benefits.

Children with ventriculoperitoneal shunts require particular attention because shunt malfunction may alter cerebrospinal fluid dynamics, increase the risk of cerebrospinal fluid leakage, and complicate the interpretation of adverse neurological symptoms after screening or implantation [37,38]. In selected situations where intrathecal catheter placement is not feasible, intraventricular baclofen has been described as a possible alternative, although evidence remains limited and this approach should be considered exceptional rather than routine [43,44].

Caregiver reliability and long-term adherence are essential components of patient selection, because ITB requires repeated refills, ongoing dose adjustments, and rapid access to specialist care in the event of suspected withdrawal or device malfunction [7,35]. For this reason, psychosocial context, travel feasibility, and family understanding of long-term treatment responsibilities should be assessed as carefully as neurological severity.

### 6.4. Contraindications

Absolute contraindications to ITB include a negative screening trial, baclofen hypersensitivity, and active local or systemic infection [7,10]. Relative contraindications include major anesthesia risk, coagulopathy, poor wound healing, severe malnutrition or inadequate soft tissue coverage, inability to safely perform future refills, and unreliable follow-up that would place the child at risk of unrecognized pump failure or baclofen withdrawal [7,10]. Severe spinal deformity does not necessarily preclude ITB, but it may substantially increase technical complexity and should be considered within individualized surgical planning.

### 6.5. Surgical Technique

Intrathecal baclofen pump implantation is typically performed under general anesthesia with placement of the pump in the abdominal wall and insertion of an intrathecal catheter via a lumbar approach under fluoroscopic guidance [35,45]. The catheter is advanced cranially to achieve an appropriate distribution of baclofen within the spinal cerebrospinal fluid, depending on the clinical pattern of spasticity.

Although the procedure is well standardized, technical considerations must be individualized in children with low body weight, severe scoliosis, previous abdominal surgery, or complex spinal anatomy. In such cases, alternative pump positioning or modified catheter routing may be required to reduce the risk of skin complications, catheter malfunction, or difficulty with pump refills [46,47,48,49].

Long-term management includes regular pump refills, dose titration, and eventual device replacement due to battery depletion in programmable systems. Because complications such as catheter dysfunction or infection may occur, implantation should be performed in centers with experience in both surgical technique and long-term multidisciplinary follow-up [7,35,50].

### 6.6. Clinical Decision-Making Algorithm for ITB in Children with CP

**Figure 1:**
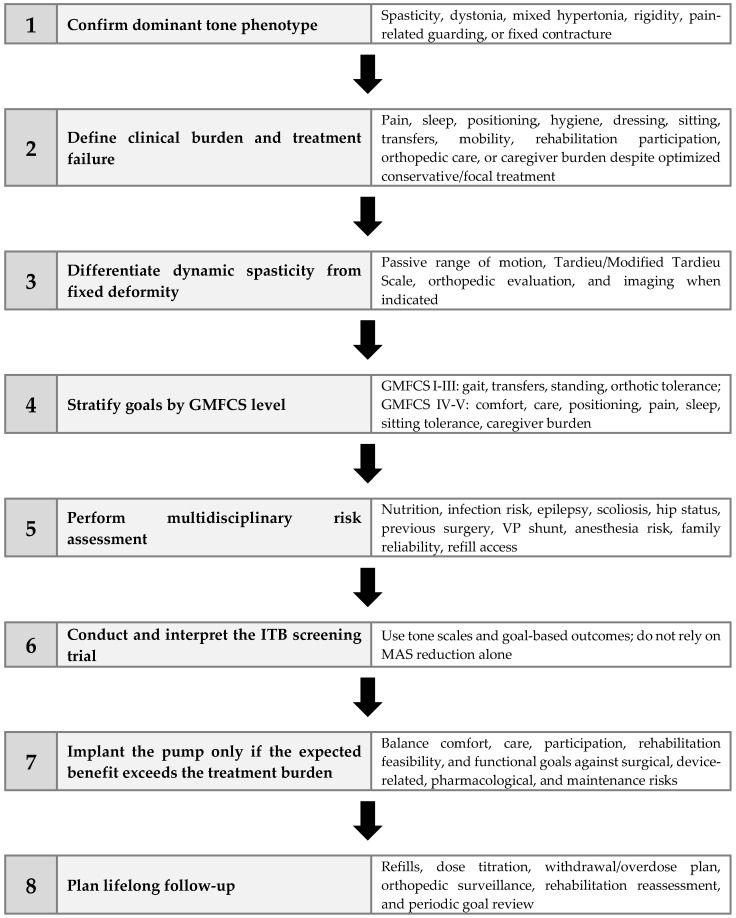
Practical decision-making algorithm for intrathecal baclofen therapy in children with cerebral palsy. The pathway emphasizes phenotype confirmation, clinical burden, differentiation of dynamic spasticity from fixed deformity, GMFCS-stratified goal setting, multidisciplinary risk assessment, screening trial interpretation, and lifelong follow-up.

A decision-making pathway for ITB (Figure 1) should begin with the determination of the dominant tone phenotype, including spasticity, dystonia, mixed hypertonia, rigidity, pain-related guarding, or fixed contracture, because these conditions differ in treatment response and prognosis [1,17,21]. The clinical burden of hypertonia should then be defined. ITB should be considered when generalized or multi-segmental hypertonia interferes with pain, sleep, positioning, hygiene, dressing, sitting, transfers, mobility, participation in rehabilitation, orthopedic care, or caregiver burden [7,10,11]. Before invasive therapy is implemented, conservative and focal treatments should be optimized, including physiotherapy, postural management, orthoses, treatment of aggravating factors, oral antispastic medication when tolerated, and botulinum toxin when focal spasticity is the dominant pattern [1,5,6]. Dynamic spasticity should be differentiated from fixed deformity using passive range-of-motion assessment, Tardieu or Modified Tardieu Scale, orthopedic evaluation, and imaging when indicated [16,22]. If fixed contracture, hip displacement, or skeletal deformity is the dominant problem, orthopedic management may be required before or instead of ITB [22,23]. Expectations should then be stratified according to GMFCS level and individualized goals. In GMFCS I–III, excessive tone reduction may impair compensatory standing or walking and should be avoided through cautious dose titration and rehabilitation monitoring [5,41]. In GMFCS IV–V, goals more often focus on comfort, care, positioning, pain, sleep, sitting tolerance, and caregiver burden rather than independent ambulation [29,40,51]. Multidisciplinary risk assessment should include nutritional status, infection risk, epilepsy, scoliosis, hip status, previous spinal or abdominal surgery, ventriculoperitoneal shunt status, anesthesia risk, family reliability, and ability to attend regular refills and urgent follow-up [7,35,37,38]. The ITB screening trial should be interpreted using both tone scales and goal-based measures [7,10]. Pump implantation is most justified when expected improvements in comfort, care, participation, rehabilitation feasibility, or selected functional goals outweigh surgical, device-related, pharmacological, and long-term maintenance risks [7,11,52]. After implantation, lifelong follow-up is required, including refill scheduling, dose titration, emergency planning for withdrawal or overdose, orthopedic surveillance, rehabilitation reassessment, and periodic review of treatment goals [33,34,35,53].

## 7. Clinical Outcomes

### 7.1. Reduction in Spasticity

The most consistent and robust effect of intrathecal baclofen therapy is a reduction in muscle tone. Multiple prospective studies and systematic reviews demonstrate significant decreases in spasticity as measured by the Ashworth Scale, Modified Ashworth Scale, and spasm frequency scores [7,10]. Recent meta-analytic data suggest an average reduction in spasticity of approximately 40% in pediatric populations treated with ITB [54]. Tone reduction is typically sustained over time with appropriate dose titration, although dose escalation may be required in some patients [55].

### 7.2. Functional Outcomes

Functional outcomes after ITB should not be treated as a homogeneous construct. In children classified as GMFCS I–III, treatment goals may include improved gait efficiency, endurance, orthotic tolerance, transfers, comfort during walking, or participation in therapy and daily activities [1,10,12]. However, this group requires particular attention because some children use increased tone as a compensatory mechanism for standing, transfers, or ambulation. Excessive reduction in tone may therefore worsen functional stability unless dosing is gradual and carefully linked to rehabilitation [5,41].

In children classified as GMFCS IV–V, expected benefits are usually different. Goals more often include improved comfort, reduced pain and spasms, easier hygiene and dressing, better sitting or lying position, improved sleep, easier transfers, reduced caregiver burden, improved tolerance of orthoses or seating systems, and facilitation of nursing and rehabilitation [11,40,51,56]. In this group, improvements in Gross Motor Function Measure scores may be modest or absent, but clinically meaningful benefit may still be substantial if comfort, care, and participation improve [10,57,58]. Therefore, outcome evaluation should include caregiver-reported and participation-focused measures rather than gross motor function alone [1,29,31].

### 7.3. Quality of Life and Caregiver Burden

Evidence suggests that ITB therapy provides clinically meaningful benefits in domains that are highly relevant to patients and caregivers, including pain reduction, sleep quality, ease of hygiene and dressing, and overall comfort [11,56]. These effects are often more pronounced in non-ambulatory children and those with severe generalized spasticity.

Improvements in caregiver burden and daily care have been consistently reported and may represent some of the most important outcomes of ITB therapy in clinical practice [40,51]. Studies incorporating participation-based outcomes also suggest benefits in social engagement and daily activities, although these effects remain heterogeneous across patient groups [57,58].

### 7.4. Complications and Long-Term Outcomes

Despite its clinical benefits, ITB therapy is associated with a substantial complication rate, reported in 20–40% of pediatric cases [35,52]. The most common complications are device-related, including catheter dysfunction, migration, obstruction, and disconnection, followed by infection and cerebrospinal fluid leakage [37,50,52].

Pharmacological complications include baclofen overdose and withdrawal, both of which may be life-threatening and require urgent recognition and management [33]. Long-term therapy is further complicated by the need for repeated pump refills, hardware replacement, and dose adjustments over time [7,53].

Although long-term tone reduction is well supported, evidence for sustained improvements in functional outcomes and participation remains limited by small sample sizes, heterogeneity of outcome measures, and lack of high-quality randomized controlled trials [10,11].

## 8. Comparison of ITB with Alternative Invasive Treatments

Intrathecal baclofen, selective dorsal rhizotomy (SDR), and botulinum toxin injections represent complementary rather than competing approaches to spasticity management in children with cerebral palsy. Their use should be guided by the distribution of spasticity, functional goals, and the overall clinical context.

Selective dorsal rhizotomy and ITB differ not only in indication but also in treatment philosophy. SDR is an irreversible ablative neurosurgical procedure that permanently reduces afferent sensory input to spinal reflex arcs. Its main advantage is durable tone reduction without implanted hardware or refill requirements, but this benefit comes at the cost of irreversibility and the need for careful selection, usually among ambulatory children with spastic diplegia, good underlying strength, and clear gait-related goals [19,59]. Inappropriately selected patients may experience weakness, sensory changes, or functional deterioration, particularly if spasticity had been contributing to compensatory standing or gait strategies [1,59].

ITB, in contrast, is reversible and programmable. Dose can be titrated, reduced, or temporarily adjusted according to growth, rehabilitation goals, intercurrent illness, sleep, pain, or evolving orthopedic problems [7,35]. This flexibility is particularly valuable in children with severe generalized spasticity, mixed tone patterns, or high care needs. However, ITB creates lifelong dependence on implanted hardware, pump refills, specialist follow-up, battery replacement, and urgent access to care in the event of withdrawal, overdose, catheter malfunction, or infection [7,34,52]. Thus, SDR and ITB should not be framed as competing procedures but as distinct strategies with different risk-benefit profiles: SDR offers permanent tone reduction in carefully selected ambulatory children, whereas ITB offers adjustable generalized tone control at the cost of device dependence and long-term maintenance.

Botulinum toxin injections are effective for focal spasticity and are widely used in outpatient settings. However, their role is limited in children with multisegmental or generalized spasticity, in which repeated injections may be insufficient to achieve global tone control [5,6].

From a clinical decision-making perspective, SDR may be preferred in selected ambulatory patients with clear gait-related goals, whereas ITB is more appropriate in children with diffuse spasticity and complex care needs. Botulinum toxin remains the first-line option for focal involvement or as an adjunct to other therapies. In this context, ITB occupies a central role in the management of severe, generalized spasticity within a comprehensive, multidisciplinary treatment strategy. All three approaches are summarized in Table 3.

## 9. Discussion

The management of spasticity in children with cerebral palsy requires an individualized, multidisciplinary approach integrating rehabilitation, pharmacological treatment, and neurosurgical interventions [1,11]. Within this framework, intrathecal baclofen (ITB) occupies a distinct role as a reversible and programmable therapy for severe, generalized spasticity that is insufficiently controlled with conservative measures [5,18].

The present review highlights that the most consistent and robust effect of ITB is reduction in muscle tone. This is supported by recent meta-analytic data demonstrating approximately 40% reduction in spasticity scores in pediatric populations [54]. This effect is well documented across studies and is typically sustained over time with appropriate dose titration [7,10]. Importantly, however, tone reduction should not be considered an endpoint in itself. In clinical practice, the primary value of ITB lies in its impact on patient-centered outcomes, including comfort, pain reduction, positioning, sleep, and ease of care [11,51]. These domains are particularly relevant for children with higher levels of motor impairment, for whom goals are often focused on care facilitation and quality of life rather than on independent ambulation [1]. Studies focusing on participation and caregiver-reported outcomes suggest sustained benefits in daily activities, social engagement, and ease of care [57,58].

In contrast, improvements in gross motor function remain variable and less predictable. Functional outcomes depend on multiple factors, including baseline motor phenotype, degree of fixed musculoskeletal deformity, timing of intervention, and alignment between treatment goals and patient capabilities [7,10]. In some ambulatory children, excessive tone reduction may even compromise compensatory mechanisms and negatively affect function [5]. These observations underscore the importance of goal-directed therapy and careful patient selection, rather than assuming uniform functional benefit from tone reduction.

From a clinical decision-making perspective, ITB should be considered primarily in children with severe, generalized spasticity associated with pain, impaired positioning, difficulties in care, or significant caregiver burden [11]. The optimal candidates are not necessarily those with the highest spasticity scores, but those in whom reduction in tone is expected to translate into meaningful improvements in daily function or quality of life. This highlights the importance of multidisciplinary assessment and realistic goal setting involving clinicians, patients, and caregivers [1].

Compared with other invasive treatment options, ITB offers several advantages, including reversibility, adjustability, and the ability to target both upper and lower limb spasticity [19]. In contrast, selective dorsal rhizotomy provides permanent tone reduction but is limited to carefully selected patients, typically ambulatory children with spastic diplegia [59]. Botulinum toxin injections remain the preferred option for focal spasticity but are less effective in generalized, multi-segmental involvement [5]. Thus, ITB occupies a complementary role within the spectrum of spasticity management strategies.

Despite its clinical benefits, ITB therapy is associated with significant risks and long-term management challenges. Device-related complications, including catheter dysfunction, infection, and the need for revision surgery, are relatively common, particularly in the pediatric population [35,52]. Additionally, baclofen overdose and withdrawal, although rare, may be life-threatening and require prompt recognition and management [33]. Long-term treatment is further complicated by the need for repeated pump refills, potential dose escalation, and hardware replacement [7]. These factors necessitate long-term follow-up in specialized centers with appropriate expertise.

The available evidence base also has important limitations. While reduction in spasticity is consistently demonstrated, data on long-term functional outcomes, participation, and quality of life remain heterogeneous [10,11]. Many studies are limited by small sample sizes, lack of randomized controlled designs, variability in outcome measures, and inconsistent follow-up durations. Moreover, standardized, patient-centered outcome measures are not uniformly applied, which complicates comparison across studies and limits the strength of conclusions regarding functional benefit.

The evidence synthesized in this review supports a practical care pathway for ITB in pediatric CP. Clinical decision-making should begin with identification of the dominant contributor to the child’s limitations, including dynamic spasticity, dystonia, pain, or fixed biomechanical deformity [1,20,21]. Treatment goals should be formulated within an ICF-based framework, separating body-function outcomes, such as tone reduction, from activity and participation outcomes, such as sitting, transfers, sleep, hygiene, mobility, comfort, and caregiver burden [17]. Expected benefits should be stratified by GMFCS level, because the clinical meaning of tone reduction differs substantially between ambulatory and non-ambulatory children [10,12,40]. The screening trial should be interpreted against individualized goals rather than as a simple pharmacological tone test [7,35]. ITB implantation should be undertaken only when the family and clinical team can commit to long-term surveillance, pump maintenance, rehabilitation adjustment, and emergency management of withdrawal or overdose [33,34]. This pathway may help translate heterogeneous evidence into reproducible clinical decision-making.

Economic considerations also play a role in treatment decision-making. ITB therapy is associated with substantial initial costs related to surgical implantation, as well as ongoing expenses for maintenance, follow-up, and device management [7]. However, these costs must be interpreted in the context of potential reductions in caregiver burden, improved comfort, and decreased need for other interventions [51].

Taken together, ITB should be viewed not as a stand-alone intervention, but as part of a comprehensive, goal-directed management strategy for spasticity in children with cerebral palsy. Its greatest value lies in carefully selected patients, in whom reduction in tone translates into meaningful improvements in comfort, care, and quality of life. Future research should focus on standardized outcome measures, long-term functional and participation outcomes, and comparative effectiveness across different treatment modalities.

## 10. Conclusions

Intrathecal baclofen is an important treatment option for children with cerebral palsy and severe generalized or multi-segmental spasticity when conservative and focal treatments are insufficient or poorly tolerated. Its most consistent benefit is reduction in dynamic tone, but its clinical value should be judged primarily by patient-centered outcomes such as comfort, pain relief, positioning, sleep, hygiene, ease of care, rehabilitation participation, and caregiver burden. Gains in gross motor function are less predictable and should be interpreted according to baseline motor phenotype, GMFCS level, degree of fixed deformity, individualized goals, and dose titration.

Successful ITB therapy requires careful differentiation between dynamic spasticity and fixed contracture, realistic counselling of families, multidisciplinary patient selection, structured screening, and lifelong specialized follow-up. Future research should prioritize standardized patient-centered outcome measures, longer-term functional and participation data, GMFCS-stratified analyses, comparative effectiveness studies across ITB, SDR, botulinum toxin, orthopedic strategies, and rehabilitation pathways. Emerging directions include smaller and more programmable pump systems, improved catheter and pump monitoring, targeted delivery strategies, digital tracking of patient-reported outcomes, and exploratory work on clinical, neurophysiological, or biological predictors of baclofen responsiveness. These developments may help refine patient selection and improve the balance between benefit, treatment burden, and long-term safety.

## Figures and Tables

**Table 1:** Comparison of the Ashworth Scale and Modified Ashworth Scale. Both scales provide a simple ordinal assessment of resistance to passive movement. The MAS adds grade 1+ to increase sensitivity for mild tone abnormalities. Because these scales do not reliably distinguish spasticity from fixed contracture or other causes of resistance, their results should be interpreted together with clinical, functional, and orthopedic assessments.

Ashworth Scale		Modified Ashworth Scale	
Grade	Description		Grade
0	No increase in muscle tone (normal or slightly decreased tone)	Minimal increase in tone at the end of the range of motion; slight resistance and release at the end range during passive movement	1
1	Slight increase in tone, giving a catch when the limb is moved in flexion or extension	Slight increase in tone, manifested by a catch followed by minimal resistance through less than half of the range of motion	1+
2	More marked increase in tone, but the limb easily flexed/extended	More marked increase in tone through most of the range, but easy movement is possible	2
3	Considerable increase in tone; passive movement is difficult		3
4	The limb is rigid in flexion or extension		4

**Table 2:** Tardieu/Modified Tardieu Scale for assessment of spasticity. The scale records the quality of muscle reaction at different stretch velocities, as well as the angles R1 and R2. The R2–R1 difference helps distinguish dynamic spasticity from fixed contracture, which is particularly relevant when evaluating children with cerebral palsy for intrathecal baclofen therapy.

Grade	Tardieu/Modified Tardieu Scale—Quality of Muscle Reaction
0	No resistance throughout passive movement.
1	Slight resistance throughout passive movement, without a clear catch at a precise angle.
2	Clear catch at a precise angle, interrupting passive movement, followed by release.
3	Fatigable clonus, lasting less than 10 s when maintaining pressure, occurring at a precise angle.
4	Unfatigable clonus, lasting more than 10 s when maintaining pressure, occurring at a precise angle.
5	Joint is immobile.
	
*Additional parameters recorded with the scale*	
**Parameter**	**Meaning**
V1	Slow passive stretch; reflects passive range of motion.
V2	Stretch at the speed of the limb falling under gravity.
V3	Fast passive stretch; used to elicit velocity-dependent spasticity.
R1	Angle of catch during fast stretch.
R2	Full passive range during slow stretch.
R2–R1	Larger difference suggests dynamic spasticity; smaller difference suggests fixed contracture.

**Table 3:** Comparison of botulinum toxin injections, selective dorsal rhizotomy, and intrathecal baclofen therapy in the management of spasticity in children with cerebral palsy.

Feature	Botulinum Toxin Injections	Selective Dorsal Rhizotomy	Intrathecal Baclofen Pump
**Type of treatment**	Pharmacological	Neurosurgical	Neurosurgical + pharmacological
**Mechanism of action**	Blocks acetylcholine release at neuromuscular junction	Selective sectioning of dorsal sensory nerve rootlets	Continuous baclofen delivery (GABA-B agonist) into CSF
**Scope of effect**	Local (targeted muscles)	Mainly lower limbs	Generalized or multi-segmental spasticity
**Duration of effect**	Temporary (3–6 months)	Permanent reduction in spasticity	Continuous while pump works
**Reversibility**	Yes	No	Yes (dose adjustable/pump removable)
**Invasiveness**	Minimally invasive	Highly invasive surgery	Moderately invasive implant
**Typical patient group**	Focal spasticity	Ambulatory children with spastic diplegia	Severe generalized spasticity
**Typical GMFCS**	I–III	I–III	III–V
**Rehabilitation**	Yes	Intensive postoperative rehab	Yes
**Maintenance**	Repeat injections required	No routine maintenance	Regular pump refills
**Potential complications**	Local weakness, injection pain	Sensory changes, weakness	Infection, catheter malfunction

## Data Availability

No new data were created or analyzed in this study. Data sharing is not applicable.

## Abbreviations

The following abbreviations are used in this manuscript:

AS	Ashworth Scale
CP	Cerebral palsy
GABA-B	Gamma-aminobutyric acid type B
GMFCS	Gross Motor Function Classification System
ITB	Intrathecal baclofen
MAS	Modified Ashworth Scale
SDR	Selective dorsal rhizotomy
